# GRANDPARENTS’ EDUCATION IS ASSOCIATED WITH GRANDCHILDREN’S EPIGENETIC AGE IN THE NGHS STUDY

**DOI:** 10.1093/geroni/igac059.875

**Published:** 2022-12-20

**Authors:** Agus Surachman, Elissa Hamlat, Anthony Zannas, Barbara Laraia, Elissa Epel

**Affiliations:** University of California, San Francisco, San Francisco, California, United States; University of California, San Francisco, San Francisco, California, United States; University of North Carolina, Chapel Hill, North Carolina, United States; University of California Berkeley, Berkeley, California, United States; University of California, San Francisco, San Francisco, California, United States

## Abstract

We leveraged information solicited from three generations (grandparents, mothers, and grandchildren) to examine the association between mothers’ childhood SES (based on grandparents’ educational attainment) and their children’s epigenetic age and whether the association was mediated by mothers’ life course socioeconomic and health-related factors. Mothers were recruited to the NHLBI Growth and Health Study when they were 9 or 10 and followed for ten consecutive years (1987-1998). Grandparents reported their highest education during the baseline interviews. Mothers were then re-contacted three decades later (ages 37-42) to participate in the National Growth and Health Study (NGHS), and health information of their youngest children (i.e., grandchildren; N = 241, ages 2-17) were collected, including their saliva samples to calculate epigenetic age. Two epigenetic ages were estimated (Horvath and Hannum), and DNA methylation age accelerations (DNAmAAs) were calculated using residuals from regressing chronologic age on each epigenetic age metrics. Mothers’ life course socioeconomic and health-related mediators included childhood BMI trajectories (from age 9 to 19), highest education level, adult health behavior, and adult c-reactive protein (CRP). Adjusted for age and sex, grandchildren with college degree grandparents showed significantly slower Horvath’s DNAmAA than those with no college degree. The association between grandparent’s education level and grandchildren’s DNAmAA was partially mediated by mothers’ life course socioeconomic and health-related factors, especially mothers’ education, health behavior, and CRP. Grandparents’ educational attainment is a critical socioeconomic context of mothers’ early rearing, and it might have a long-lasting impact on their grandchildren’s epigenetic marker.

